# The optics of introspection: a two-axis model of self-reflective distortion

**DOI:** 10.3389/fpsyg.2026.1936705

**Published:** 2026-10-01

**Authors:** Swapan Samanta

**Affiliations:** Indoriv Clinical Research Centre, Kolkata, India

**Keywords:** Aberration unmasking principle, construct validity, contemplative psychology, directional calibration bias, introspection, mediating field, self-knowledge, self-reflection

## Abstract

What kind of instrument do we use when we examine ourselves—and how much of what we see is shaped by that instrument rather than by what we actually are? I propose here that self-reflective cognition operates through a system subject to two separable classes of aberration. *Directional calibration bias* (Axis I) describes a stable functional tendency to systematically inflate (positive bias) or deflate (negative bias) the self-image across domains. The *mediating field* (Axis II) describes the temporal, interpersonal, cultural, and perspectival conditions through which self-information passes before reaching awareness. We argue that these axes are separable: reducing mediation does not correct calibration bias, and correcting calibration bias does not dissolve the mediating field. From this separability we derive the *Aberration Unmasking Principle*: that reducing one class of distortion may reveal a pre-existing distortion previously hidden beneath it. The model treats introspective distortion as continuously distributed—everyone introspects, but the optical parameters vary across the population, and most people fall in a range where distortion is present but subclinical. We outline decision rules for classifying phenomena onto each axis, provide a conceptual formalization, propose sample items for the Introspective Aberration Inventory (IAI), and discuss heuristic convergences with contemplative traditions. The framework generates falsifiable predictions and constitutes the first empirically tractable component of a broader research agenda in what we term Metaphysical Optics.

## 1 Introduction

Consider two people who each believe they are more generous than they actually are. In the first case, the inflation is pervasive—the person also overestimates their intelligence, attractiveness, and moral standing. Take away their social circle, change their cultural context, and the inflation persists. It is not anchored to any particular experience or relationship. It is a property of how they evaluate themselves, full stop.

In the second case, the overestimation of generosity is specific. It traces back to a childhood in which small kindnesses were lavishly praised, to a cultural milieu that rewards performative giving, to a social group that never challenged the self-image. Place this person in a different relational context with different feedback, and the self-image shifts. The distortion is real, but it is not structural—it is the accumulated residue of particular conditions through which the person came to know themselves.

Both people score identically on a self-report measure. Both overestimate their generosity. But the mechanism differs, the prognosis differs, and the corrective intervention should differ. The first person needs their evaluative apparatus restructured; the second needs the accumulated deposits of experience identified and dissolved. Current psychological frameworks—from the introspection illusion (Pronin, 2009) to self-enhancement research (Taylor and Brown, 1988) to depressive realism (Alloy and Abramson, 1979)—do not formally distinguish these two kinds of error. They document that introspection fails, sometimes in specific ways. But they do not ask a prior question: when introspection fails, is the failure a property of the instrument itself, or a property of the conditions through which the information passed?

We propose such a distinction here.

### 1.1 Definitions

Because the terms in this domain are used inconsistently across traditions, I specify the following definitions for this paper. *Self-knowledge* is an individual's representation of their own psychological states, traits, capacities, and standing—the totality of what a person takes themselves to know about themselves. *Introspection* is the directed examination of one's own mental states and processes, following Schwitzgebel (2019). *Self-reflection* is the broader evaluative process in which an individual takes some aspect of themselves as the object of examination—encompassing explicit propositional self-assessment (Morin, 2011), metacognitive monitoring (Flavell, 1979), and affective self-appraisal (Silvia and Duval, 2001). *Self-study* serves as the encompassing term for the full process, from the initial availability of self-information through its representation and interpretation. I use these terms throughout with these specific meanings.

Table 1 summarizes the key constructs introduced in this paper.

**Table 1:** Key constructs and definitions.

Construct	Definition
Directional calibration bias (Axis I)	Stable functional tendency for self-representation to deviate toward inflation or deflation relative to criterion-referenced standards
Mediating field (Axis II)	The temporal, interpersonal, cultural, and perspectival conditions through which self-information passes before representation
Criterion-relative calibration	Minimal systematic directional error relative to specified external standards (behavioral, predictive, interpersonal)
Aberration unmasking principle	Reducing one class of distortion may increase the observability of a pre-existing distortion previously hidden by it
Optical inclusion criterion	An optical construct enters the framework only if it identifies a distinct mechanism not reducible to existing constructs and generates a discriminating prediction
Epistemic image displacement	The distance between the apparent source assigned to an experience and its best-supported generative source

### 1.2 The status of the optical analogy

#### 1.2.1 A necessary clarification

When I speak of mirrors, curvature, and films, I am not claiming that the brain contains an optical system. The framework is a structural analogy—a mapping between the well-developed vocabulary of physical optics and a set of psychological phenomena that have been documented independently but never integrated into a common architecture. The analogy is constrained by the Optical Inclusion Criterion: an optical principle enters the framework only if it identifies a distinct psychological mechanism, cannot be reduced to an existing construct, and generates a prediction or argument that would not arise without it. Analogy as a method of theory construction has a long history in science (Gentner, 1983; Gentner and Markman, 1997), and its legitimacy depends not on whether the source domain literally describes the target but on whether the structural mapping is productive—whether it organizes phenomena, generates predictions, and reveals connections that would otherwise remain invisible.

Three claims of this paper are independent of the optical terminology: (1) that introspective distortion has at least two separable dimensions; (2) that reducing one may expose the other; and (3) that different dimensions respond to different interventions. These claims would survive the complete removal of every optical term from the paper. The optical framework provides the organizing vocabulary and the generative analogy from which constructs such as Epistemic Image Displacement are derived. The vocabulary is dispensable; the structural claims are not.

### 1.3 What the optical framework adds

The natural question is: why optics? What does this vocabulary provide that existing models of cognitive bias, self-enhancement, and social mediation do not? Three things.

#### 1.3.1 First, the Aberration unmasking principle

No existing framework in the introspection literature predicts that reducing one form of distortion can *increase the observability* of another. This prediction follows specifically from the two-axis architecture and the separability claim—it does not follow from any single-factor model of introspective error, however sophisticated.

#### 1.3.2 Second, the differential-intervention prediction

The two-axis model predicts that interventions targeting mediation (e.g., identifying and dissolving accumulated cultural or interpersonal deposits) will have different effects on the two axes than interventions targeting calibration (e.g., systematic feedback training, self-other discrepancy reduction). No existing model generates this specific dissociation.

#### 1.3.3 Third, the continuous-spectrum prediction

The OI model treats introspective distortion as *parametric* rather than categorical. Everyone introspects—it is constitutive of human consciousness. But the parameters of each person's introspective system (the curvature of the evaluative apparatus, the density and composition of the mediating field) vary continuously. Most people fall in a central range where the bias is mild, the mediation is moderate, and the resulting self-image is serviceable. We do not all walk around in states of narcissistic inflation or depressive collapse. But neither do we see ourselves with perfect clarity. The question is not whether introspection is accurate or inaccurate—it is where on a continuous, multi-dimensional distribution a given individual falls, and what the intervention implications of that location are.

This replication-crisis framing also matters. When a discipline treats two separable dimensions as one, effect sizes will fluctuate across samples as a function of the unmeasured dimension. If calibration bias and mediating-field effects are genuinely separable, then any measure that conflates them will produce unstable results across populations—not because the phenomenon is unreliable but because the construct is underspecified (cf. Eronen and Bringmann, 2021; Borsboom et al., 2021).

### 1.4 Scope and novelty

No existing framework in psychology, philosophy, or contemplative science formally distinguishes the directional orientation of the introspective apparatus (Axis I) from the mediating conditions through which self-information is transmitted (Axis II), demonstrates their separability, or derives the interaction effects—particularly the Aberration Unmasking Principle—that follow from treating them as separable dimensions. The framework integrates Western empirical psychology (the introspection illusion, depressive realism, self-enhancement research) with Buddhist and Vedāntic accounts of self-knowledge into a common structural vocabulary, without reducing either tradition to the other. It generates falsifiable predictions, proposes a conceptual assessment architecture, and constitutes the first empirically tractable component of a broader research agenda in what I term Metaphysical Optics.

The paper is structured as follows. Section 2 reviews the existing landscape as a demonstration of why heterogeneous introspective error demands a two-axis model. Section 3 presents the model—decision rules for axis classification, the two axes, their separability, the possibility of dependence, named descriptive profiles, and a conceptual formalization. Section 4 introduces the Aberration Unmasking Principle and its alternative explanations. Section 5 discusses heuristic convergences with contemplative traditions. Section 6 introduces an Image-Formation Taxonomy. Section 7 briefly notes the larger research agenda. Section 8 outlines empirical predictions, a null model, and a proposed assessment architecture. Section 9 discusses limitations.

## 2 The evidence for heterogeneous introspective error

I do not claim that prior scholars have treated introspective error as literally unitary. The literature recognizes confabulation (Nisbett and Wilson, 1977), self-enhancement (Taylor and Brown, 1988), depressive distortion (Beck, 1967), the introspection illusion (Pronin, 2009), and other modes of failure. What is missing is not the catalog but the architecture—a formal distinction between errors that originate in the evaluative apparatus and errors that originate in the conditions of transmission.

### 2.1 Self-enhancement as directional bias

Taylor and Brown's (1988) review documented pervasive positive bias in self-evaluation among non-depressed individuals: people rate themselves as above average, overestimate control, and maintain unrealistically optimistic expectations. This is not a domain-specific effect that varies with what is being evaluated. It is a baseline orientation of the evaluative apparatus—a consistent tilt. The depressive realism findings (Alloy and Abramson, 1979; Moore and Fresco, 2012) complement this: mildly depressed individuals make more accurate self-evaluations, suggesting that the non-depressed default is not neutral but positively biased. Neuroimaging evidence points to differential fronto-temporal activation during self-serving vs. non-self-serving attributions, suggesting the bias is implemented at a level deeper than verbal report.

In the framework proposed here, self-enhancement is an instance of positive calibration bias—a concave tilt of the evaluative apparatus that inflates across domains.

### 2.2 Confabulation and mediation

Nisbett and Wilson's (1977) classic studies, Wilson (2002) adaptive-unconscious research, and Pronin (2009) introspection illusion demonstrate something different. People construct plausible but incorrect explanations for their own behavior, drawing on cultural scripts, temporal reconstruction, and situational cues rather than accessing actual causal processes. The resulting self-image may be inflated, deflated, or simply irrelevant to the actual process it claims to describe.

This is not a directional bias. The self-information has passed through layers of cultural expectation, temporal reconstruction, and narrative convention that have reshaped it before it reached awareness. The confabulated explanation is filtered and reconstructed—not systematically tilted in one direction. It is a mediating-field effect.

### 2.3 What prior frameworks each capture—and miss

A brief comparison clarifies why the two-axis model is not merely a relabeling of existing work.

Cooley's (1902) looking-glass self identifies one type of mediating condition—the interpersonal—and treats it as the primary mechanism of self-knowledge formation. Cooley has no account of directional calibration. Lacan's (1949/2006) mirror stage identifies one direction of calibration—the concave—and treats it as universal. Lacan has no mediating field and no convex pole. The introspection illusion (Pronin, 2009) documents the general unreliability of introspection but does not distinguish types of unreliability. Depressive realism (Alloy and Abramson, 1979) identifies one region of the calibration continuum—the mildly depressed as closer to plane—but does not address mediation. Self-discrepancy theory (Higgins, 1987) distinguishes ideal-self and ought-self discrepancies but does not distinguish whether the discrepancy arises from the evaluative apparatus or from accumulated mediating conditions. Metacognitive monitoring (Flavell, 1979; Koriat, 2007) addresses calibration of self-assessment but not the mediating conditions through which self-information is transmitted. Predictive processing and Bayesian self-models (Seth, 2013; Limanowski and Blankenburg, 2013) provide computational architectures for self-modeling but do not distinguish structural bias from mediating conditions.

Freud (1914/1957) came closest to the present distinction: his account of the ego-ideal as an internalized evaluative structure, and his distinction between self-observation and ego-defense, gestures toward a separation between the instrument of evaluation and the conditions of its operation. Jaspers (1913/1997) recognized limits on self-understanding that are not reducible to motivated distortion. Taylor (1989) argued that the sources of the self—the moral frameworks through which we interpret ourselves—condition self-knowledge in ways that are not simply biases. Each of these thinkers identified something real. None formalized the two-axis architecture or derived the interaction effects that follow from it.

### 2.4 Why this is not dual-process theory

Readers acquainted with dual-process theories (Kahneman, 2011; Evans and Stanovich, 2013) may wonder whether the two-axis model is a variant—System 1 as mediation, System 2 as calibration. It is not. Dual-process theories distinguish modes of processing: fast and automatic vs. slow and deliberate. The OI model distinguishes sources of distortion. Calibration bias can operate in either mode—a person can have a fast, automatic self-enhancement tendency or a slow, deliberate one (the carefully constructed flattering narrative). Similarly, cultural mediation can operate automatically (unexamined absorption of evaluative schemas) or deliberatively (conscious identification with a cultural self-image). The two axes cut across the processing-mode distinction.

## 3 The optics of introspection

### 3.1 Decision rules for axis classification

Before presenting the axes, I address a concern that any two-axis model must confront: how do we decide, in a given case, which axis a distortion belongs to? I propose three diagnostic criteria, acknowledging that ambiguous cases will exist and that the criteria are empirical hypotheses subject to revision.

#### 3.1.1 Cross-domain consistency

If a distortion is consistent across unrelated self-evaluation domains—a person inflates their intelligence, their kindness, their attractiveness, and their professional competence roughly equally—the primary source is likely calibration bias. If the distortion is domain-specific (generous self-image of kindness but accurate self-assessment of intelligence), it is more likely mediated by domain-specific experiential deposits.

#### 3.1.2 Persistence after context removal

If the distortion persists after the hypothesized mediating condition is removed or changed—the person continues to inflate after leaving the cultural context that reinforced inflation—the residual distortion is attributable to calibration. If it dissolves, it was mediation.

#### 3.1.3 Informant convergence

If multiple informants from different relational contexts agree that the person systematically over- or underestimates themselves, this suggests calibration bias (the distortion is visible across observers and contexts). If different informants in different contexts report different discrepancies, this suggests mediating-field effects operating differently in different relational settings.

These criteria do not resolve every case. Chronic mediating conditions can, over developmental time, shape calibration—I discuss this possibility explicitly in Section 3.4. But the criteria provide an operationalizable starting point. Two worked examples illustrate their application.

#### 3.1.4 Example 1

A person overestimates their professional competence. Investigation reveals they also overestimate their intelligence, kindness, attractiveness, and athletic ability with roughly equal magnitude (cross-domain consistency). The overestimation persists after changing employers, social circles, and cultural context (persistence). Colleagues, family members, and strangers all report the same discrepancy (informant convergence). Provisional classification: calibration bias (Axis I). Contrast: a second person overestimates professional competence specifically, traces the self-image to a career in which early successes were disproportionately praised, and shows accurate self-assessment in non-professional domains. The overestimation diminishes when they move to a context with different feedback. Provisional classification: interpersonal and temporal mediation (Axis II).

#### 3.1.5 Example 2 (mixed case)

A person chronically underestimates their social likability. The underestimation originated in childhood social exclusion—a specific mediating history (Axis II). But 20 years of living with this self-image have gradually shaped the evaluative apparatus itself: the underestimation now extends to non-social domains and persists in welcoming social environments. The mediation has, over developmental time, produced calibration. This is the internalization pathway—a genuine challenge to the model that the Separability Thesis accommodates by distinguishing developmental origin from functional present-tense architecture. The clinical prediction is specific: this person's calibration bias will show partial sensitivity to mediation-targeted interventions (since it retains traces of its mediating origin), whereas purely structural calibration bias will not.

### 3.2 Axis I: directional calibration bias

Calibration bias (the abbreviated form I use throughout, after this formal introduction as *directional calibration bias*) describes a stable functional tendency of the self-evaluative apparatus to produce representations that are systematically shifted in a positive direction (concave), maintained near criterion-relative accuracy (plane), or systematically shifted in a negative direction (convex).

#### 3.2.1 Two clarifications

First, when I call calibration bias a “structural” or “stable” tendency, I am identifying a *functional theoretical construct*—not a fixed biological entity or an immutable personality trait. Calibration bias is consistent enough to be measurable and consequential, but it is modifiable through therapeutic restructuring, contemplative practice, or sustained corrective feedback. “Structural” here means functionally consistent across domains and over time, not hardwired. The analogy is with attachment working models—stable but revisable.

Second, when I speak of ‘accuracy' at the plane position, I mean *criterion-relative calibration*: minimal systematic directional error relative to specified external standards—behavioral predictions, informant agreement, longitudinal stability, or ecological validity. I am not claiming access to “the self as it truly is.” A person's self-assessment of their generosity is well-calibrated if it predicts their actual giving, is corroborated by diverse informants, and remains stable across assessment contexts.

#### 3.2.2 At the concave pole

Narcissistic self-enhancement (Morf and Rhodewalt, 2001), grandiosity (Pincus and Lukowitsky, 2010), the self-serving bias (Campbell and Sedikides, 1999). Robins and John (1997) showed that narcissists displayed increased self-admiration when allowed to observe themselves from an external perspective—evidence that the inflation is a property of the evaluative apparatus, not a motivational overlay.

#### 3.2.3 At the convex pole

The depressive cognitive triad (Beck, 1967), pathological self-criticism (Blatt, 1995), the impostor phenomenon (Clance and Imes, 1978), and what Gilbert (2000) describes as an internal self-to-self relationship of contempt. Convex bias must be distinguished from accurate modesty. The plane mirror yields a genuinely modest image when the person is genuinely modest. Convex bias produces systematic underestimation regardless of what the criteria show.

### 3.3 Axis II: the mediating field

Independent of calibration, the self-information that reaches awareness has passed through conditions that alter it in ways the concave–plane–convex continuum does not capture. I use the term *mediating field* rather than “surface filming” because the operations involved go beyond deposition and coating—they include transmission, absorption, selective filtering, scattering, refraction, and attenuation.

I identify four primary classes of mediating condition, each producing a characteristic pattern of transformation. Whether these four constitute a single latent dimension or four correlated subfactors is an empirical question the IAI validation study is designed to answer; I present them here as conceptually distinguishable components within the broader Axis II construct.

#### 3.3.1 Temporal mediation

The fading affect bias (Walker et al., 2003), nostalgia (Sedikides et al., 2008), rosy retrospection (Mitchell et al., 1997), and affective forecasting errors (Wilson and Gilbert, 2005). The past self is seen through a warm filter; the future self through a lens of exaggerated consequence.

#### 3.3.2 Interpersonal mediation

Cooley (1902) reflected appraisals, intensified through attachment dynamics (Bowlby, 1969). Early caregivers deposit the densest layers.

#### 3.3.3 Cultural mediation

Bourdieu's (1977) doxa—assumptions so shared they are invisible. Gender, caste, class, and professional identity scripts each constitute a distinct cultural film. Foucault's (1988) “technologies of the self” can be read as a genealogy of cultural mediation: each era deposits its own residue on the introspective surface while claiming to clean it.

#### 3.3.4 Perspectival mediation

Construal level theory (Trope and Liberman, 2010)—psychological distance shifts construal from concrete to abstract, producing different self-images at different vantage points without either being inherently more accurate.

### 3.4 The separability thesis

A terminological note to prevent slippage. Throughout this section I use three related but distinct terms. *Separability* is a conceptual claim: the constructs can be independently defined. *Partial independence* is an empirical prediction: they will show discriminant validity in measurement. *Interaction* is a derived prediction: their joint effects differ from the sum of their individual effects. Conceptual separability does not imply statistical independence, and interaction does not contradict separability.

The central structural claim is that calibration bias and the mediating field are separable. I choose this word carefully—not “orthogonal,” which claims too much, and not merely “distinct,” which claims too little. Separability means three things:

- **The separability proposition**: the two axes are conceptually distinct and can be independently defined, operationalized, and measured.
- **The partial independence proposition**: they will show discriminant validity in psychometric assessment. (The strong form predicts low correlation; the weak form predicts discriminant but correlated dimensions).
- **The interaction proposition**: they interact in clinically and contemplatively significant ways—most importantly through the Aberration Unmasking Principle.
- A reviewer might reasonably ask: if the axes interact, how can they be independently measured? The answer is straightforward and has parallels throughout science. Temperature and humidity are independently measurable—each can be assessed with a separate instrument that does not require knowledge of the other variable. They also interact significantly—perceived thermal comfort depends on both. Independent measurability and interaction are not contradictory; they are precisely what makes a two-dimensional model more informative than a one-dimensional one.

### 3.5 On the possibility of dependence

An honest theory must confront the strongest objection to its own architecture. Three forms of dependence are plausible:

#### 3.5.1 Causal dependence

Chronic exposure to certain mediating conditions may, over developmental time, produce calibration bias. A child raised in relentless criticism may develop a structurally convex apparatus—the mediation has shaped the curvature. But the resulting calibration bias persists even after the mediating condition is removed, which is why the separability thesis has clinical significance.

#### 3.5.2 Developmental dependence

Attachment theory (Bowlby, 1969) suggests that early interpersonal mediation shapes not only the mediating field but the evaluative apparatus itself. If so, the two axes share developmental precursors while remaining functionally separable in adulthood. The claim is not that they have independent origins—it is that they can be independently measured, independently addressed, and that fixing one does not automatically fix the other.

#### 3.5.3 Statistical dependence

I expect partial correlation. The strong OI hypothesis predicts *r* < 0.30; the weak form predicts discriminant but correlated dimensions. Both predict that a correlated two-factor model will outperform a single-factor model. If the axes prove highly correlated (*r* > 0.70), the conceptual distinction survives but its practical value diminishes.

### 3.6 The two-dimensional state space

Crossing the axes generates six identifiable regions (Figure 1). I offer provisional descriptive labels for these regions—not as established categories but as heuristic names that may prove useful if the two-axis structure is empirically confirmed. They are placeholders, not diagnoses.

**Figure 1:**
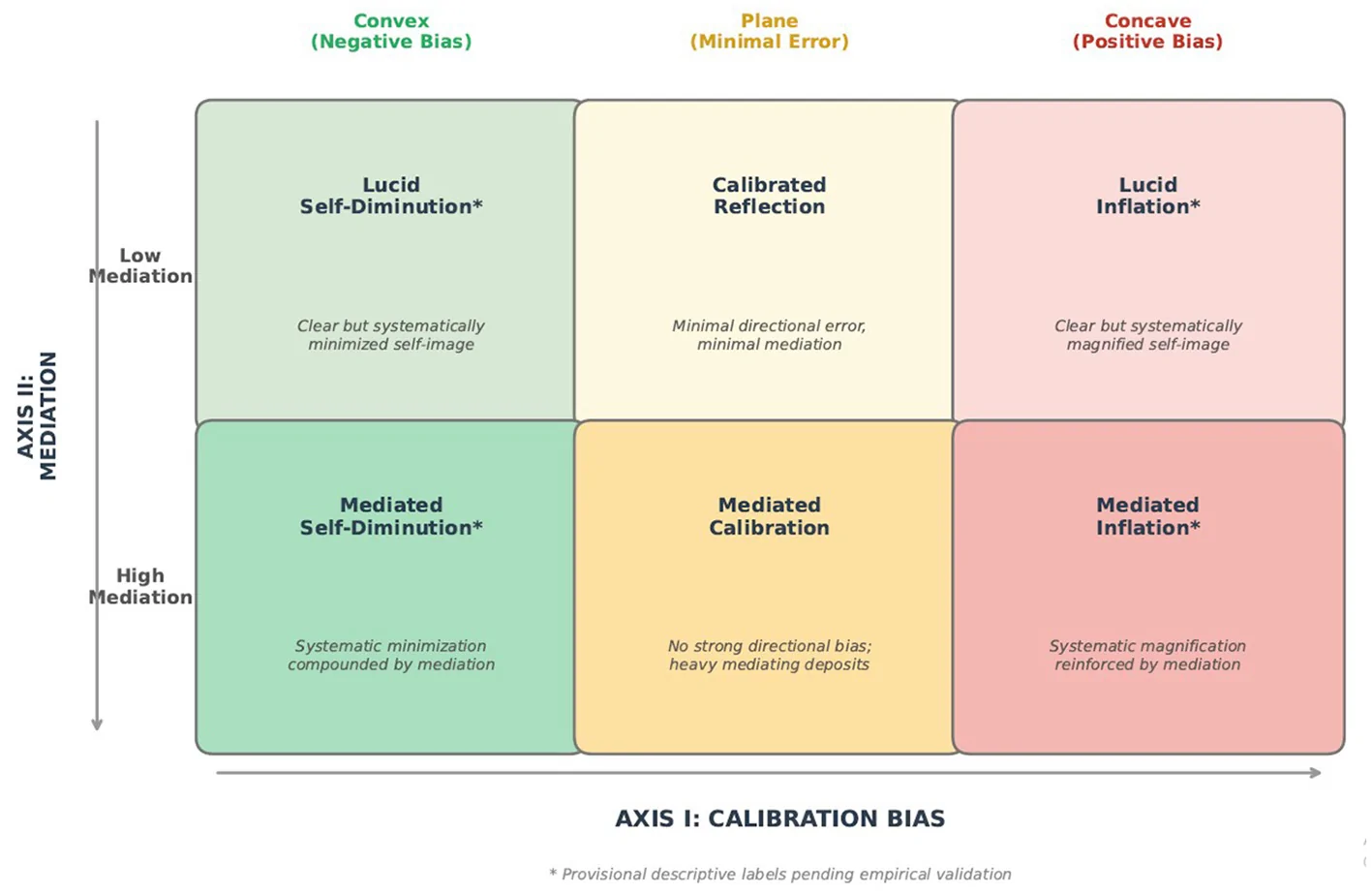
The two-dimensional introspective state space. Columns represent calibration bias (Axis I). Rows represent mediating-field density (Axis II). Asterisks indicate provisional labels pending validation.

Two of these profiles deserve attention because they are counterintuitive. LucidInflation^*^ (low mediation, concave bias) describes someone who has achieved substantial clarity—they are not deluded by cultural conditioning or interpersonal dependency—but their evaluative apparatus is structurally inflationary. They see clearly, and what they see is a magnified self. This profile is immediately recognizable in contemplative contexts as the advanced practitioner whose deconditioning has exposed, rather than resolved, underlying narcissism (Trungpa, 1973; Welwood, 2000). Its mirror image, *LucidSelf*−Diminution^*^ (low mediation, convex bias), describes someone who has stripped away protective mediating layers and confronts a structurally diminished self-image without the softening effect of those layers.

### 3.7 Conceptual formalization

I offer a minimal formalization—not a computational model, but a conceptual equation and causal structure that can guide future formal development.

#### 3.7.1 Conceptual schema

*R* = *f(T, C (T), M(T, context), O)*, where R is the self-representation, T is the target, C is the calibration function applied to the target, M is the mediating-field transformation (which depends on both the target and the context), and O represents the observation conditions. The separability thesis claims that C and M are functionally distinguishable components of this equation—that varying C while holding M constant produces different R values than varying M while holding C constant.

The causal structure runs: illumination → Target availability → Mediating-field transformation → Calibration transformation → Self-representation → Interpretation and stabilization → (recursion: consequences feed back into mediation and illumination for the next cycle). A directed acyclic graph could be drawn, though the recursive loop introduces cyclicality that complicates static representation. I leave full computational formalization to future empirical work and offer here the theoretical grammar that such formalization would implement.

## 4 The Aberration unmasking principle

The model's strongest derivative contribution: *reduction of one class of introspective distortion may increase the observability, behavioral expression, or measurable effect of a pre-existing distortion that was previously obscured by it—without necessarily increasing the magnitude of that distortion itself* .

### 4.1 The last clause matters

The Unmasking Principle does not predict that de-mediation creates calibration bias. It predicts that de-mediation reveals calibration bias that was already present but masked. The distortion does not become stronger; it becomes more visible (Figure 2)—to the individual, to observers, and to measurement instruments. The distinction is operationalizable: unmasking predicts increased self-other discrepancy on calibration-relevant dimensions after a de-mediation intervention, without a corresponding increase on calibration-irrelevant control dimensions.

**Figure 2:**
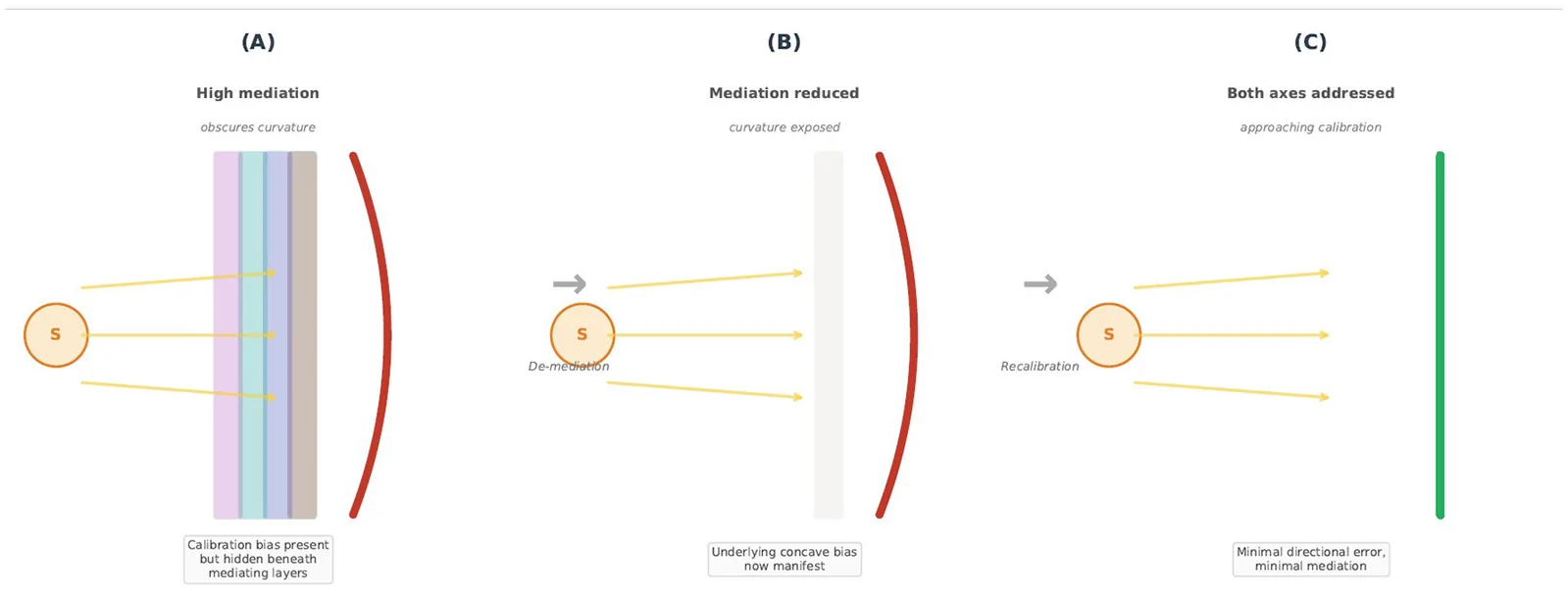
The Aberration unmasking principle. **(A)** High mediation obscures underlying concave curvature. **(B)** De-mediation exposes the calibration bias. **(C)** Both axes addressed.

This principle applies beyond contemplative contexts. Spiritual narcissism after retreat practice (Welwood, 2000). Surfacing of grandiosity after depression treatment (Pincus and Lukowitsky, 2010). Identity crisis after exiting a relationship that heavily mediated self-image. In each case, the pattern is the same: a mediating layer was removed, and the underlying calibration—previously invisible beneath it—became manifest.

Alternative explanations must be taken seriously. The apparent unmasking could reflect: (a) increased self-focus—the intervention simply directs more attention inward, producing general salience rather than axis-specific revelation; (b) response-criterion shift—the participant becomes more willing to report unflattering self-perceptions; (c) demand characteristics—participants infer what the intervention is supposed to reveal; (d) confidence reduction—reduced mediation lowers certainty rather than exposing calibration; (e) regression to the mean in high-mediation participants selected for extreme scores. The experimental design proposed in Section 8.2 addresses these through an active control group (controlling for self-focus), signal-detection indices for response bias, calibration-irrelevant control dimensions (if unmasking is axis-specific, it should not appear on unrelated dimensions), and multiple assessment points to distinguish genuine unmasking from statistical artifact.

## 5 Heuristic convergences with contemplative traditions

### 5.1 An epistemological disclaimer before proceeding

The comparisons in this section are *philosophical analogies*—structural parallels between the OI framework and certain contemplative traditions. They are not claims that these traditions anticipated the present model, not comprehensive interpretations of the traditions cited, and not empirical equivalences. I distinguish three levels: historical interpretation (what a thinker meant in context), philosophical analogy (structural similarity between frameworks), and empirical psychological theory (testable claims about mechanism). The following discussion operates exclusively at the second level.

The Shenxiu–Huineng exchange in the Platform Sutra (McRae, 2000) dramatizes precisely the question the OI model raises: does purification of mediating conditions exhaust the problem of self-apprehension? Shenxiu's verse describes continuous de-mediation—polishing the mirror. Huineng's counter-verse questions whether the mirror's own structure might be part of the problem. The OI model offers a vocabulary for this structural ambiguity without claiming that either master anticipated it.

The Vedāntic concept of *sākṣin* (witness-consciousness; Gupta, 1998) provides a heuristic comparison with the plane-mirror ideal—awareness that neither magnifies nor diminishes. Whether this condition is an ontological given (Vedānta) or a regulative ideal approachable through corrective practice (the OI model) is a question the present framework deliberately leaves open.

The zero-aberration condition can be further connected to the formal model of perpetual self-transcendence I have proposed elsewhere (Samanta, 2026a), which defines liberation as the inability to fix one's identity in any achieved position. Within the OI framework, this maps onto the plane condition understood not as a static flatness but as the absence of any stabilized curvature—the evaluative apparatus does not lock onto a single self-image. The Somatic Codification Hypothesis (Samanta, 2026b) extends this into the bodily domain, proposing that contemplative recognition has a measurable somatic signature on the interoceptive–exteroceptive axis. For the OI model, this suggests that the mediating field includes bodily conditions—muscular holdings, postural habits, visceral patterns—that are not typically recognized as mediating self-knowledge but that may function as some of the densest and least visible mediating layers. The neurophysiological integration model I have proposed for kundalini-related practice (Samanta, 2026c) documents multi-domain biomarker changes during intensive contemplative work. Within the OI framework, such integration may correspond to progressive de-mediation across somatically encoded layers—and the documented adverse effects of rapid integration (disorientation, identity instability, affective dysregulation) are consistent with the Aberration Unmasking Principle: accelerated de-mediation may expose pre-existing calibration bias that slower, graduated practice would surface more manageably. The Equilibrium Geometry of Flourishing (Samanta, 2026d) provides a further connection: its non-compensability axiom and Burden Law show that gain without proportional Vairāgya (autonomy-as-non-seizure) increases duratva rather than reducing it. Within the OI framework, calibration bias is itself a form of seizure—the evaluative apparatus locked onto a fixed directional orientation—and the Burden Law finds a structural parallel in the prediction that richer self-information through a curved mirror yields sharper but more misleading images, not greater accuracy.

## 6 An image-formation taxonomy

Not all departures from calibration are of the same kind. I propose a graded taxonomy, ordered by decreasing target constraint (Figure 3):

**Figure 3:**
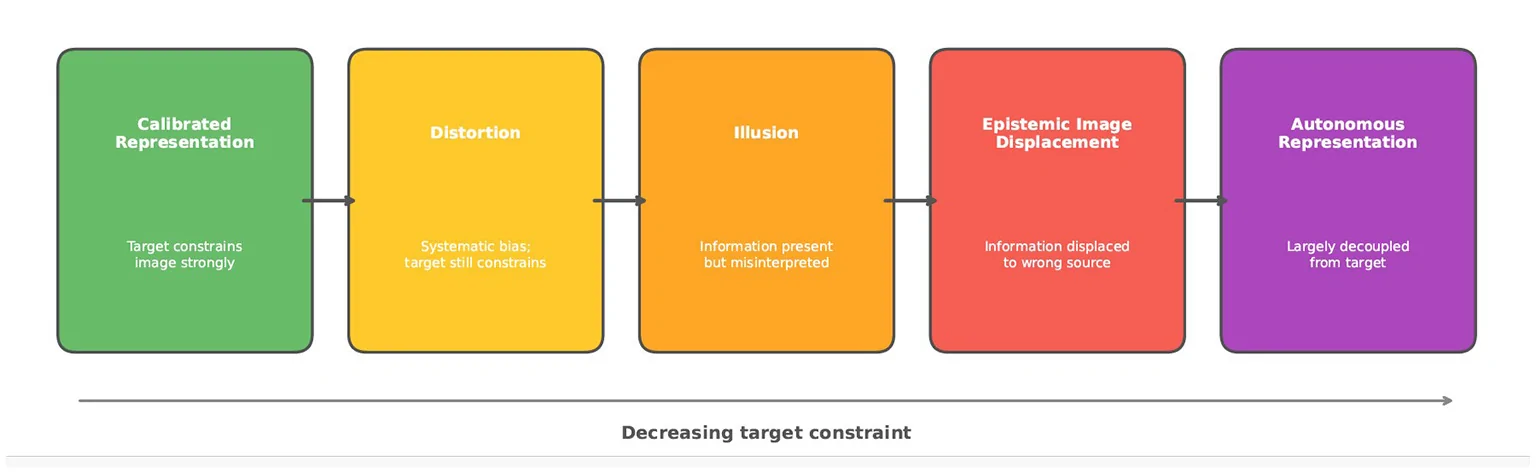
The image-formation taxonomy, from calibrated representation (high target constraint) to autonomous representation (low target constraint).

*Calibrated representation*: the self-image tracks the target with minimal systematic error. *Distortion*: systematic directional bias but the target still constrains the image. *Illusion*: genuine information present but systematically misinterpreted. *Epistemic Image Displacement* (for which I retain the illustrative label ‘mirage'): genuine experiential information attributed to a source, identity, or cause where the generative source does not reside—success interpreted as destiny, guilt attributed to inherent corruption when the source is an internalized parental voice. *Autonomous representation*: self-images largely decoupled from target constraints, maintained and elaborated independently of evidence. I deliberately avoid equating this with clinical psychosis, which is multi-determined.

## 7 The larger research agenda

The two-axis model abstracts the two most empirically tractable components from a broader theoretical architecture. A companion paper in preparation develops additional constructs—the illumination function, the mirror ownership problem, the recursive optical circuit—each satisfying the Optical Inclusion Criterion. The present paper establishes the foundation.

## 8 Empirical predictions and assessment

### 8.1 The null model

A two-axis model must be tested against a simpler alternative. The null model predicts a single introspective-distortion factor. If a one-factor model provides a better fit to the data, the OI model is disconfirmed. The OI model predicts at minimum two discriminable dimensions, differential sensitivity to specific interventions, and unmasking interaction effects.

### 8.2 Predictions

#### 8.2.1 Prediction 1 (discriminant structure)

A correlated two-factor model (calibration and mediating-field density) will fit significantly better than a single-factor model. I compare one-factor, correlated two-factor, bifactor, and multidimensional models, letting the data adjudicate.

#### 8.2.2 Prediction 2 (differential intervention)

Interventions targeting mediation (structured exercises addressing temporal reconstruction, interpersonal reflected appraisal, cultural schema identification, perspectival reframing) will reduce Axis II scores more than Axis I scores. Interventions targeting calibration (systematic feedback discrepancy training, self-other agreement exercises) will reduce Axis I scores more than Axis II scores.

#### 8.2.3 Prediction 3 (Aberration unmasking)

A concrete design: screen participants using the IAI Mediating Field subscale. Assign those above the 75th percentile randomly to an 8-session structured de-mediation intervention (targeting temporal and interpersonal mediating conditions through guided reflection) or waitlist control. Pre- and post-intervention measures: IAI Calibration Bias Scale, Narcissistic Personality Inventory (Raskin and Terry, 1988), self-other agreement indices (Vazire, 2010). The unmasking prediction is confirmed if the intervention group shows increased self-other discrepancy on calibration-relevant dimensions relative to controls, without a corresponding increase on calibration-irrelevant control dimensions. Expected effect: medium (*d* ≈ 0.4–0.6). Alternative explanation to rule out: general self-focus increase rather than axis-specific unmasking.

#### 8.2.4 Prediction 4 (mediating-field specificity)

Different mediating conditions respond to different class-matched interventions. Temporal mediation responds to prospective-retrospective calibration; interpersonal mediation to attachment-informed relational work; cultural mediation to critical consciousness-raising; perspectival mediation to construal-level training.

#### 8.2.5 Prediction 5 (neural dissociability, exploratory)

Dissociable multivariate neural signatures for calibration bias and mediating-field effects. Framed as exploratory to avoid reverse-inference problems.

### 8.3 A proposed assessment architecture

The proposed IAI is envisioned as one component of a multimethod assessment architecture—not a standalone diagnostic instrument. It has not been empirically validated; what follows is a conceptual blueprint. The proposed IAI comprises a Calibration Bias Scale and a Mediating Field Scale (the latter with temporal, interpersonal, cultural, and perspectival subscales). Sample items:

**Calibration bias scale—Concave pole:** “When I succeed at something, I usually attribute it primarily to my own abilities rather than circumstances.” “I tend to think I am better than most people at things that matter to me.”

**Calibration bias scale—Convex pole:** “When I succeed, I usually feel it was mainly due to luck or external help.” “I often feel that other people are more competent than I am, even when evidence suggests otherwise.”

**Mediating field scale—Temporal subscale:** “My view of my past self is strongly colored by how I feel right now.”

**Interpersonal subscale:** “I find it difficult to form a view of myself that is independent of what important people in my life seem to think of me.”

**Cultural subscale:** “My sense of my own worth is heavily shaped by my culture's definition of success.”

**Perspectival subscale:** “My self-assessment changes dramatically depending on the situation I am in.”

An informant-report version enables computation of self-other discrepancy scores—a non-paradoxical measure of calibration bias following the logic of Vazire's (2010) SOKA model. Validation plan: Phase 1—item generation and content validation through expert review; Phase 2—exploratory factor analysis in a university sample (target *N* ≥ 300); Phase 3—confirmatory cross-validation in an independent sample with convergent and discriminant validity assessment.

## 9 Discussion

### 9.1 What this paper claims—and does not

The OI model is a theoretical framework for organizing heterogeneous forms of introspective distortion. It is not a neural theory, a clinical diagnostic, or a metaphysical proof. Its value lies in its organizational power, its capacity to generate novel predictions—particularly the Aberration Unmasking Principle—and its ability to connect bodies of research that have been studied independently. No clinical application is recommended on the basis of this theoretical framework alone. The clinical implications described here identify directions for future translational research, not current practice recommendations.

### 9.2 Limitations

The model's central claims remain hypotheses. The IAI has not been piloted. The predictions await testing. This is a theory paper, and its proposals will stand or fall on empirical evidence.

The two axes may prove more correlated than the model predicts. Developmental interactions between chronic mediation and calibration formation are plausible and would complicate application without invalidating the conceptual distinction.

The optical analogy has limits. Not every optical phenomenon generates a useful psychological construct. The Optical Inclusion Criterion constrains overextension, but judgment is always required about when an analogy illuminates and when it merely decorates.

The philosophical references—Rorty (1979), Lacan, Foucault, the Platform Sutra, the Vedāntic sākṣin—are selective readings in the service of structural comparison, not comprehensive interpretations. Scholars of these traditions may find the readings reductive. I acknowledge this limitation while maintaining that the structural parallels are genuinely informative.

The paper draws primarily on Western and South/East Asian traditions. Sufi, Indigenous, and African philosophical accounts of self-knowledge are not integrated. This is a limitation that future theoretical work should address.

## 10 Conclusion

When we look inward, we are not looking through clear glass. We are using an instrument—and that instrument may be curved, and the medium it looks through may be clouded. These are different problems. Cleaning the medium does not straighten the instrument, and straightening the instrument does not clean the medium. This simple structural insight organizes a surprisingly wide range of disconnected phenomena: self-enhancement and depressive realism, spiritual narcissism and the impostor syndrome, the Shenxiu-Huineng debate and the clinical observation that treating depression sometimes reveals grandiosity.

The contribution is not the optical metaphor—metaphors are easy. The contribution is the formal separation of two dimensions of introspective distortion, the derivation of the Aberration Unmasking Principle from their separability, and the continuous-spectrum model that explains why the same universal process—introspection—produces dramatically different outcomes in different people. Not because some people introspect and others do not, but because the parameters of the introspective system vary continuously, and most of us fall in a range where the distortion is present, real, and consequential, but not extreme enough to force itself into view.

## Data Availability

The original contributions presented in the study are included in the article/supplementary material, further inquiries can be directed to the corresponding author.
